# Genetically diverse *Pseudomonas aeruginosa* populations display similar transcriptomic profiles in a cystic fibrosis explanted lung

**DOI:** 10.1038/s41467-019-11414-3

**Published:** 2019-07-30

**Authors:** Adrian Kordes, Matthias Preusse, Sven D. Willger, Peter Braubach, Danny Jonigk, Axel Haverich, Gregor Warnecke, Susanne Häussler

**Affiliations:** 1 Institute for Molecular Bacteriology, TWINCORE, Centre for Experimental and Clinical Infection Research, Hannover, 30625 Germany; 2 Institute for Molecular Bacteriology, Helmholtz Centre for Infection Research, Braunschweig, 38124 Germany; 3 Institute of Pathology, Hannover Medical School, Hannover, 30625 Germany; 4 Biomedical Research in Endstage and Obstructive Lung Disease Hannover (BREATH), German Center for Lung Research (Deutsches Zentrum für Lungenforschung [DZL]), Hannover, 30625 Germany; 5 Department of Cardiothoracic, Transplant and Vascular Surgery, Hannover Medical School, Hannover, 30625 Germany

**Keywords:** Bacteria, Clinical microbiology, Pathogens

## Abstract

Previous studies have demonstrated substantial genetic diversification of *Pseudomonas aeruginosa* across sub-compartments in cystic fibrosis (CF) lungs. Here, we isolate *P. aeruginosa* from five different sampling areas in the upper and lower airways of an explanted CF lung, analyze ex vivo transcriptional profiles by RNA-seq, and use colony re-sequencing and deep population sequencing to determine the genetic diversity within and across the various sub-compartments. We find that, despite genetic variation, the ex vivo transcriptional profiles of *P. aeruginosa* populations inhabiting different regions of the CF lung are similar. Although we cannot estimate the extent to which the transcriptional response recorded here actually reflects the in vivo transcriptomes, our results indicate that there may be a common in vivo transcriptional profile in the CF lung environment.

## Abstract (web summary)

*Pseudomonas aeruginosa* displays substantial genetic diversification across sub-compartments in cystic fibrosis (CF) lungs. Here, Kordes et al. show that, despite genetic variation, the ex vivo transcriptional profiles of *P. aeruginosa* populations are similar across five different areas in an explanted CF lung.

## Introduction

The environmental bacterium *Pseudomonas aeruginosa* can be found in a large variety of terrestrial and aquatic habitats. A pronounced metabolic versatility confers to this broad ecological success^1^. *Pseudomonas aeruginosa* is also an opportunistic pathogen that plays a dominant role as the causative agent of severe acute as well as chronic infections^2–4^. In cystic fibrosis (CF) patients *P. aeruginosa* adopts a biofilm mode of growth and persists in the respiratory tract, despite even intensified antimicrobial therapy^5,6^. Ongoing inflammation and changes in the structure and function of the affected lungs produce strong selective pressures. Nevertheless, single *P. aeruginosa* clones infect CF patients for life^7,8^.

It has recently been demonstrated that there is large within-patient pathogen population diversity in the CF environment^9–13^. Diversity increases the potential of whole populations to adapt to changes in environmental conditions^14^. However, we are only at the beginning to understand the link between diversity and community structure and function^15–19^. In addition to genetic diversity^9,20–22^, the transcriptional response contributes to bacterial adaptation to a challenging environment, such as that encountered in the CF lung during a chronic infection progress. There have been two large previous studies on the transcriptional profile of *P. aeruginosa* during a chronic infection of the CF lung^23,24^. Those studies clearly demonstrated that *P. aeruginosa* faces antibiotic, oxidative, and osmotic stress in the microenvironment of a CF lung and controls quorum-sensing-regulated virulence factor production in order to overcome the challenges.

Here we isolated *P. aeruginosa* from five different areas in the upper and lower airways of an explanted CF lung and applied colony-re-sequencing and deep population-sequencing approaches to determine the genetic diversity of individual isolates within and across the various lung sub-compartments. We then analyzed whether the genetic variability of the isolates from the sub-compartments is reflected in differences in their ex vivo gene expression profile. Our results indicate that clinical isolates adopt a gene expression profile that is largely independent of the genetic background of the populating isolates in the different sub-compartments. Interestingly, the *P. aeruginosa* ex vivo gene expression profiles of an explanted lung correlates well with the previously recorded CF sputum profiles and seem to reflect a gene expression profile that is fixed by commonly identified within-host *P. aeruginosa* mutations.

## Results

### *Pseudomonas aeruginosa* sub-populations in an explanted CF lung

**Fig. 1:**
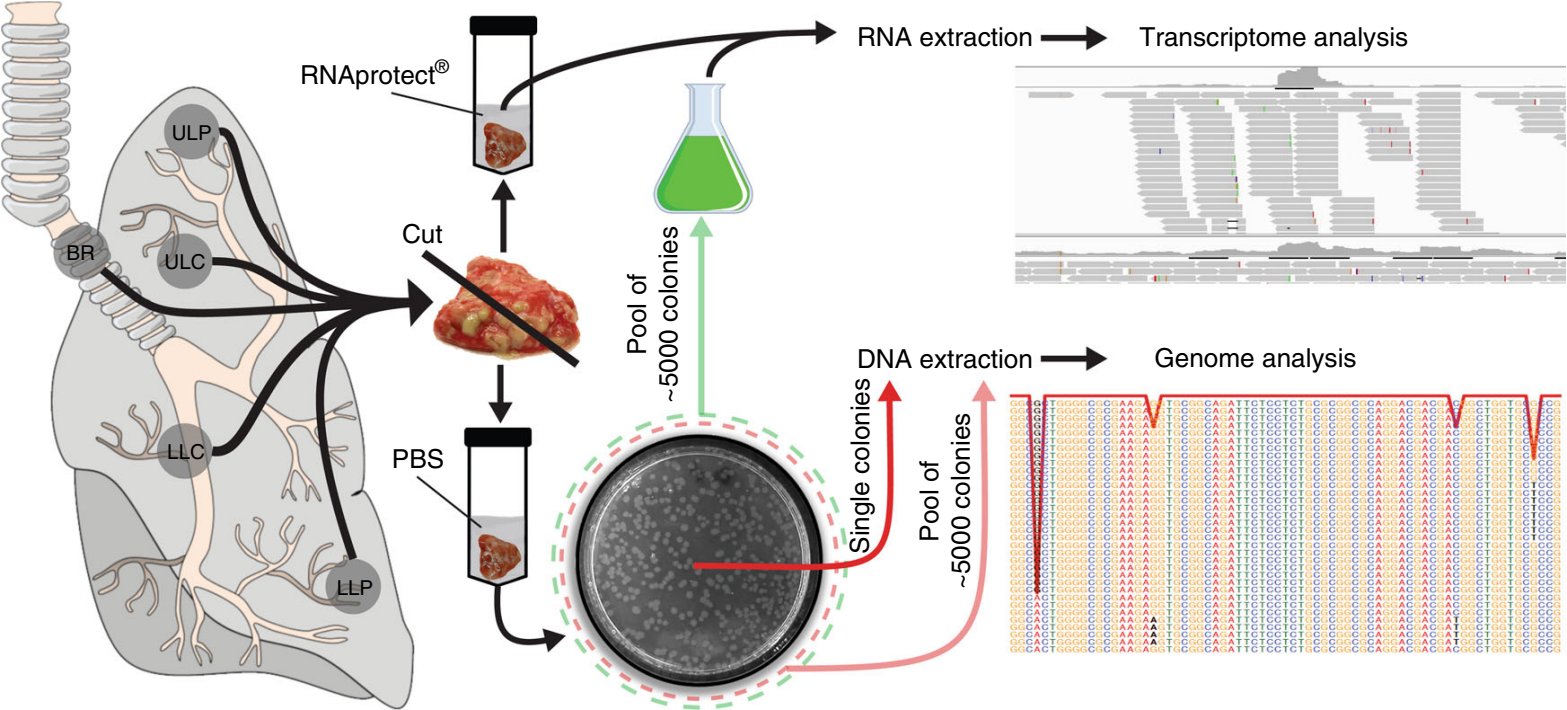
Collection of regional *P*. *aeruginosa* populations from an explanted cystic fibrosis (CF) lung. Tissue from five different compartments of an explanted CF lung was sampled (main bronchus = BR; upper lobe central = ULC and peripheral = ULP; lower lobe central = LLC and peripheral = LLP). One part of the tissue was homogenized in the presence of RNAprotect for ex vivo RNA-sequencing (RNASeq) and the other part was streaked on selective agar plates to recover single *P. aeruginosa* isolates (after homogenization in phosphate-buffered saline (PBS)). Pools of ~5000 single colonies (green dashed circle) from the five sub-compartments were cultivated in rich medium conditions to record in vitro transcriptome profiles by RNASeq. DNA was extracted from overnight cultures of single isolates and from cell pellets of pools of agar grown colonies (red dashed circle)

**Fig. 2:**
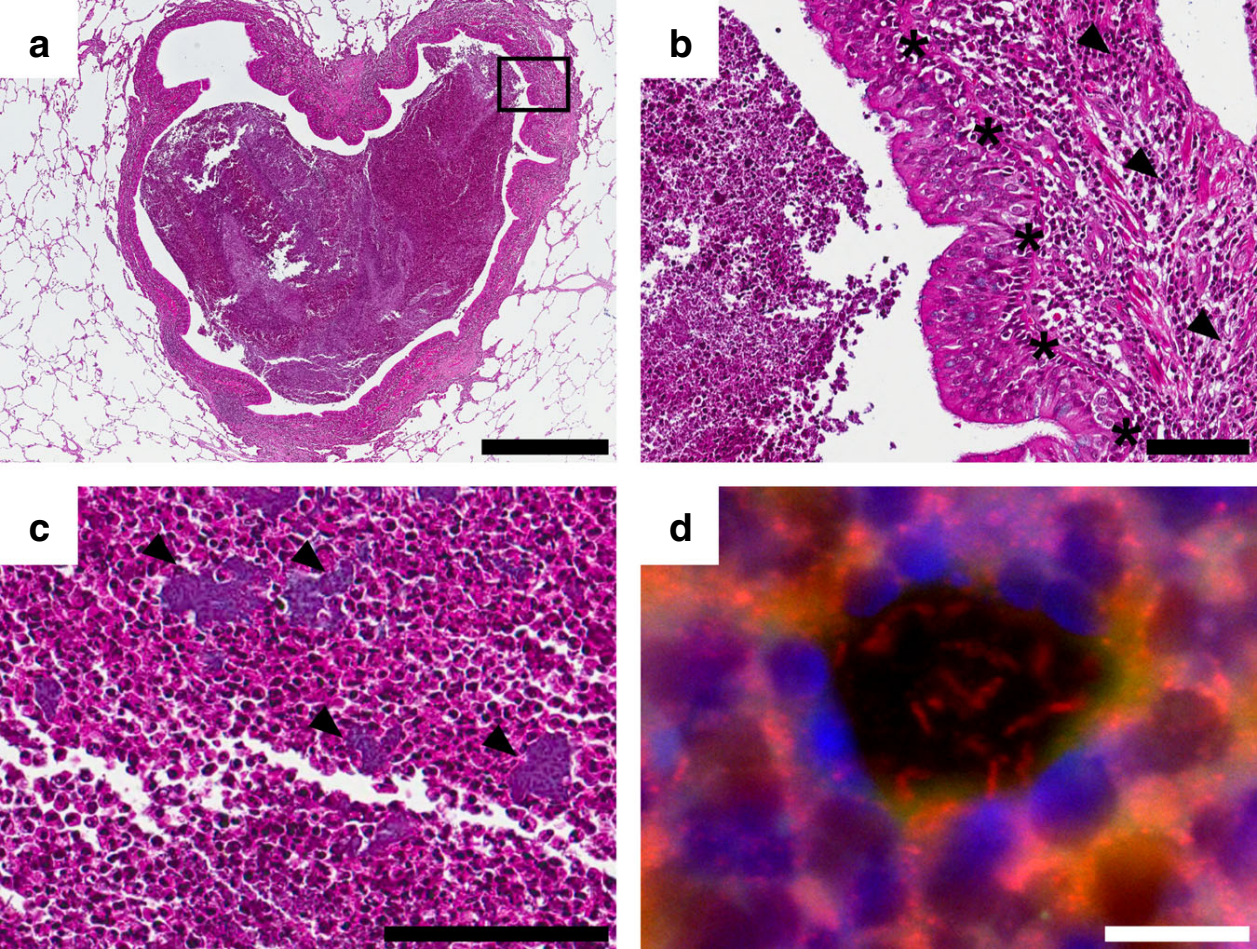
Histological analysis of the cystic fibrosis (CF) lung. The hematoxylin–eosin-stained tissue sections (**a**–**c**) show lung parenchyma with a dilated conducting airway (bronchiectasis) filled with mucopurulent secretions (**b** detail of **a**). The airways retain their typical lining with ciliated columnar epithelium (asterisk). Reserve cell and goblet cell hyperplasia as well as scarring and chronic inflammation (arrowhead) of the bronchial wall can be observed. **c** Bacterial colonies (arrowhead) are found in the airway lumen mucopurulent secretions. **d** Residing bacteria are identified as *P. aeruginosa* by application of a species-specific fluorescence in situ hybridization. Scale bars of 1000 µm (**a**), 100 µm (**b**, **c**), and 10 µm (**d**) are included

We aimed to explore the successful adaptation of *P. aeruginosa* to the environment of the CF lung. We therefore sampled and sequenced *P. aeruginosa* isolates from different regions of an explanted CF lung and at the same time performed extensive ex vivo transcriptome analysis. Tissue sampling of the left lung lobe of an explanted CF lung was performed within 30 min after transfer of the lung away from the surgical field. Samples were taken at five sites: main bronchus (BR), upper lobe central (ULC) and peripheral (ULP), and lower lobe central (LLC) and peripheral (LLP). To recover individual *P. aeruginosa* isolates, tissue samples were streaked on *P. aeruginosa*-selective agar plates, whereas for ex vivo transcriptional profiling all samples were homogenized in RNAprotect directly after removal (Fig. 1). The lung was explanted from a DeltaF508 homozygous CF patient, who underwent lung transplantation at the age of 35. The patient had been chronically infected with *P. aeruginosa* for more than 20 years. Light microscopy assessments of lung samples were conducted to observe histopathological changes (Fig. 2). Hematoxylin–eosin staining of lung parenchyma showed a dilated conducting airway (Fig. 2a), which was further characterized by scarring and chronically inflamed tissue (Fig. 2b). The airway lumen was filled with mucopurulent secretions and bacterial micro-colonies (Fig. 2c). In these bacterial colonies, *P. aeruginosa* could be identified by fluorescence in situ hybridization (FISH) (Fig. 2d, Supplementary Fig. 1).

### Genetic compartmentalization of *P. aeruginosa* in the CF lung

**Fig. 3:**
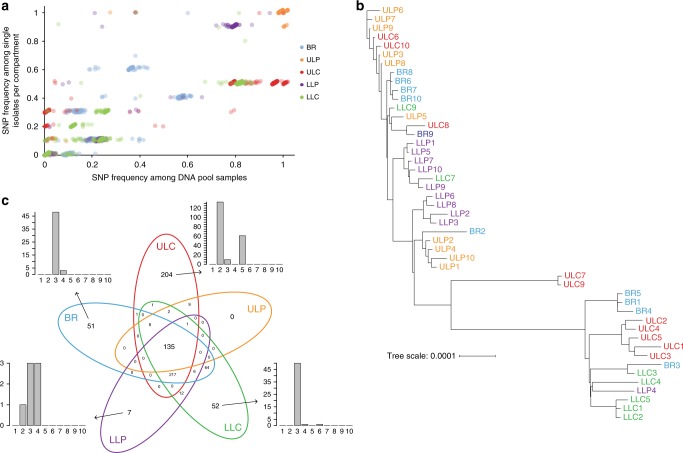
Genome sequencing of single isolates and pools. **a** The single-nucleotide polymorphism (SNP) frequency in 10 single isolates per compartment was correlated to the SNP frequencies in the pools (mean of two replicates) of the respective compartment. The overall linear association between the detection of SNPs in single genome vs. pooled genome sequencing was high (Pearson’s correlation coefficient *r* = 0.924, adjusted *p* value ≤0.001). Slight jitter was added to increase visibility (up to 2% change). **b** Genomes from single isolates were de novo assembled and the phylogenetic tree was created based on a multi-FASTA alignment of all of the core genes using PRANK. The phylogenetic tree was visualized with the iTOL online tool. Color code of the lung isolates indicates from which sub-compartment they were recovered: main bronchus = blue, upper lobe central = red, upper lobe peripheral = orange, lower lobe central = green and lower lobe peripheral = purple. **c** Venn diagram of the distribution of the 770 identified SNPs in the isolates across the five lung compartments. The bar plots indicate the number of the compartment-specific SNPs that are shared by a number of individual isolates among the overall 10 sequenced isolates from each of the compartment. Source data are provided as a Source Data file

We randomly picked ten bacterial colonies from the *P. aeruginosa*-selective agar plates, which have been inoculated with tissue samples of the five different lung regions. DNA was isolated from subcultures of those 50 isolates and the genomes were sequenced by the use of Illumina technology at high sequencing depth (~110× genome coverage). We also sequenced the DNA isolated from cell pellets of pools of ~5000 agar-plate-grown isolates that have been recovered from the five individual regions with an ~490× coverage depth. The genomes were de novo assembled and the sequence information of the core genes was used to construct a phylogenetic tree. The clinical isolates clustered with the PAO1 reference strain, so that we aligned the sequencing reads to the PAO1 genome (GenBank accession number NC_002516.2) in order to detect single-nucleotide polymorphisms (SNPs) in the individual isolates. We also determined the relative fraction of sequencing reads harboring SNPs in the five pools. A compartment-wise comparison of the detection of genetic diversity in the single isolate sequencing vs. the deep pool sequencing approach is shown in Fig. 3a. Overall, there was a strong and significant linear association between the detection of SNPs in single genome vs. pooled genome sequencing as detected by Pearson’s correlation (*r* *=* 0.924). We found the highest correlation coefficient in the ULP region (*r* *=* 0.992) and the lowest in the BR region (*r* *=* 0.778). Thus, as has been described before^10^, the pooled sequencing approach reliably detected the polymorphisms and largely mirrored the relative frequency of SNPs in the *P. aeruginosa* isolates as determined by single genome sequencing. Our results also demonstrate that the sequence variation of ten selected isolates showcases the genetic diversity within the pools recovered from the various sub-compartments.

Three isolates, from the lower central region, showed elevated SNP numbers, indicating a hypermutator phenotype. Indeed, we found nonsynonymous mutations in the *mutS* and the *mutY* gene in these isolates^25^, so that they were excluded from further analysis. Phylogenetic clustering based on a multi-alignment of core genes of the remaining 47 *P. aeruginosa* isolates (Fig. 3b) indicated that the *P. aeruginosa* isolates that were sampled from the same region were more closely related to each other than they were to isolates from distant regions. For further confirmation, we analyzed the distribution of the SNPs across the clinical isolates. Among all 47 sequenced *P. aeruginosa* isolates, we found SNPs at overall 770 positions (Fig. 3c). In general, there was a higher number of SNPs in the isolates recovered from the central regions as compared to the peripheral regions. In all, 482, 578, and 498 SNPs were detected in the isolates of the BR, ULC, and LLC, respectively, whereas only 155 and 378 SNPs were found in the ULP and LLP, respectively (Fig. 3c). A median of 26 SNPs were detected among two isolates. However, the median number of sequence variations between two isolates of the same compartment was significantly lower (12 SNPs) than between different compartments (271 SNPs) (adjusted *p* value ≤0.001, Mann–Whitney *U* test). Our results corroborate the previous finding that *P. aeruginosa* isolates that have been sampled from the same region exhibit an overall more similar SNP profile than *P. aeruginosa* isolates from different regions, a phenomenon that has been described as genetic compartmentalization^9^.

### Directional selection of genetic variations

**Fig. 4:**
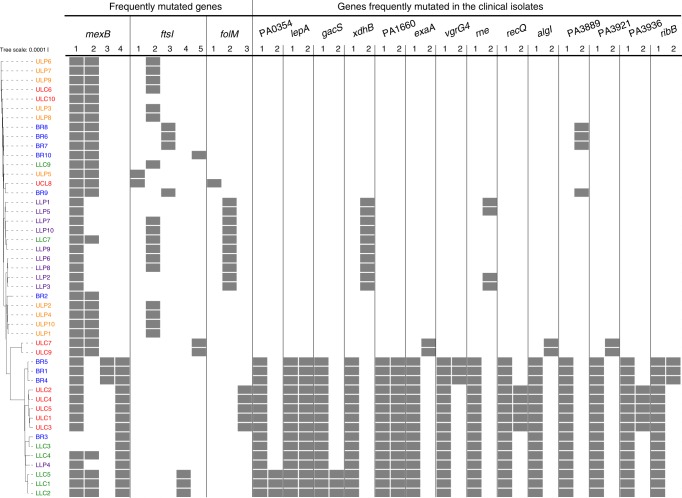
Directional selection of genetic variations. Genes with multiple mutations and the corresponding clinical isolates harboring these mutations are shown. The top three genes harboring more than two mutations were *mexB*, *ftsI*, and *folM*. Furthermore, genes are listed with two mutations and those occurred in at least 15 isolates. The gray squares indicate the presence of the mutation in the respective clinical isolate. Color code of the lung isolates indicates from which sub-compartment they were recovered: main bronchus = blue, upper lobe central = red, upper lobe peripheral = orange, lower lobe central = green, and lower lobe peripheral = purple

We next sought to evaluate whether there are mutations in the clinical isolates that are positively selected in the CF lung during the infection process. The genetic signatures of positively selected mutations are expected to be found commonly in the infecting organisms. We found the same inactivating *lasR* as well as *mexZ* mutations in all 47 clinical isolates. Both mutations have previously been shown to be positively selected for in the CF lung^26–28^. Additionally, three genes (*ftsI*, *mexB*, and *folM*) harbored dominant SNPs that were—in various combinations—repeatedly identified in a majority of the clinical isolates (Fig. 4). Of note, the presence of SNPs in those genes was not restricted to a single compartment of the lung, indicating that the environment of the chronically infected CF lung selects for these sequence variations. The *ftsI*, *mexB*, and *folM* genes are linked to antibiotic resistance and thus might have been under selective pressure. However, these mutations might also be hitchhiking with other mutations that are strongly selected for. The MexAB-OprM efflux pump is known to confer to multidrug resistance^29^, the penicillin-binding protein 3, encoded by *ftsI* is a target of aztreonam^30^, and *folM*, which encodes a dihydromonapterin reductase, plays a role in folate biosynthesis. Loss-of-function mutations in *folM* were shown to contribute to co-trimoxazole resistance^31,32^.

In addition to those three frequently mutated genes, Fig. 4 lists genes that harbored two SNPs in at least 15 isolates. Recovery of those isolates tended to be biased towards distinct regions, indicating that the individual sub-compartments might select for different alleles. For instance, mutations in *gacS*, *algI*, or *exaA* were found mainly in the central regions, although it must be taken into account that overall more SNPs and a greater heterogeneity was found in the central regions.

### Expression of a common ex vivo transcriptional profile

We have previously shown that clinical *P. aeruginosa* isolates share very similar transcriptional profiles if cultured under the same environmental conditions, despite a broad variation in their phylogenetic origin^33^. In order to explore whether and to what extent genetic heterogeneity would shape the transcriptional profile in the environment of the CF lung, we recorded the ex vivo transcriptional profile of the *P. aeruginosa* populations from the various CF lung sub-compartments. We sequenced Illumina RNA libraries and obtained ~37 million raw reads per sample (mean). After depletion of ribosomal RNA (rRNA) reads during library preparation, ~850,000 high-quality reads (mean) mapped to the PAO1 reference genome, indicating that ~97% of all reads represented human transcripts. Of those 850,000 high-quality reads, ~79% represent messenger RNA (mRNA) reads and ~20% rRNA reads. RNA-sequencing (RNASeq) does not only give information on the quantity of reads that map to the individual genes of the reference genome, but generation of complementary DNA (cDNA) also provides information on sequence variations within the mapped reads. In order to explore whether the ex vivo gene expression profiles reflects gene expression of the entire population within one compartment, we correlated the mutation frequencies in our ex vivo cDNA samples (RNASeq) to those that were found by the genome sequencing approach. The ULP and LLC region showed high Pearson’s correlation coefficients of the mutation frequencies in cDNA as compared to the DNA sequencing pools (*r* *=* 0.83–0.95, Supplementary Fig. 2). The correlation coefficients decreased with lower cDNA coverage, so that the correlation coefficients in the LLP and LLC samples were lower (Supplementary Fig. 2). Nevertheless, our data clearly indicate that the individual sub-clones within one compartment contribute to the gene expression profile and their relative contribution to the gene expression reflects their abundance.

Sequencing of the genomes of *P. aeruginosa* isolates recovered from five different sub-compartments revealed genetic diversity (Fig. 3b, c). To test whether this genetic diversity was reflected in the ex vivo transcriptional profiles of the isolates, we compared transcriptional profiles from the bronchus, central (ULC, LLC), and peripheral (ULP, LLP) region, as well as from the bronchus, upper (ULC, ULP), and lower (LLC, LLP) region. EdgeR-based differential gene expression analysis did not detect any differentially expressed gene between the respective compartments. To account for the transcriptional noise, we increased sensitivity of the test using a analysis of variance (ANOVA)-like test and identified 15 differentially expressed genes between bronchus, central, and peripheral regions (Supplementary Table 1). To further evaluate whether there were subtle compartment-specific gene expression profiles, we focused on significant functional enrichment of gene subsets, which exhibited an at least two-fold differential gene expression between sub-compartments. The analysis was performed on the top 100 genes, ordered according to their *p* value (all of which did not pass the significance filter). Functions (biological processes, Gene Ontology (GO))^34,35^ of these genes were defined to be significantly enriched using the hypergeometric test (R function phyper, adjusted *p* value ≤0.05). Indeed, we found four GO terms, in which genes that were up-regulated in the peripheral lung compartments (ULP, LLP) were significantly enriched in comparison to the central compartments (ULC, LLC) and four GO terms in which genes that were down-regulated were significantly enriched (Supplementary Table 2). Pyoverdine biosynthesis genes, genes involved in siderophore transport and cell surface receptor signaling, were obviously expressed at higher levels in the lung periphery and in the upper lung lobes, whereas genes of the arginine deaminase pathway were involved in response to stress, as well as nitrogen and nitrate compound metabolic processes were expressed at lower levels in the lung periphery. Genes that were up-regulated in the upper lung (ULC, ULP) compared to the lower lung (LLC, LLP) compartments were enriched in additional four GO terms, including heme and iron ion transport (Supplementary Table 2). These results indicate that there are subtle adaptations in gene expression profiles, which could reflect genetic differences of the inhabitant populations and/or differences in the lung environment. Thereby, it seems that the upper lung and the lung periphery share environmental cues.

### In vitro expression of variant transcriptional profiles

**Fig. 5:**
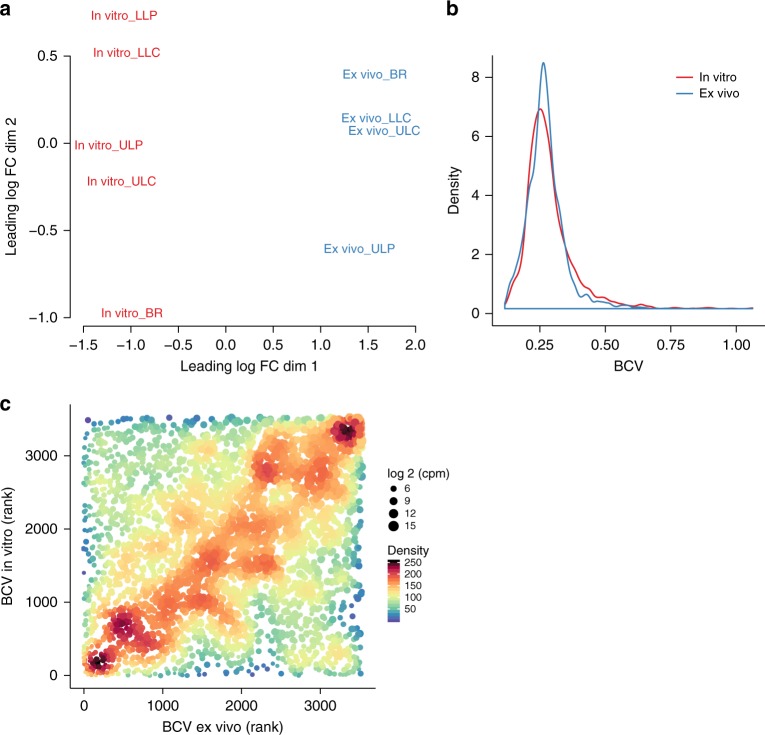
Variances of gene expression in vitro and ex vivo. To reduce the influence of sequencing depth on variance, sample reads were subsampled to the same level and filtered (counts per million (cpm) in all samples ≥5). Distribution of normalized reads was comparable in all samples after data processing (Supplementary Fig. 3a). In addition, subsampling did not affect distribution of normalized reads and also the number of remaining genes after filtering was similar (Supplementary Fig. 3a). The ex vivo LLP transcriptional profile has been removed prior analysis because <500,000 reads were obtained. **a** Multidimensional scaling plot of ex vivo and in vitro samples. **b** Density plot of the biological coefficient of variation (BCV) for the ex vivo and in vitro transcriptional profiles. The median values were the same for both conditions (ex vivo: 0.265 (95% confidence interval (CI) = 0.2633–0.2668); in vitro: 0.267 (CI = 0.2643–0.2693); CI’s were calculated using the basic bootstrap method (5000 iterations). Different data processing did not have a major effect on the outcome (Supplementary Fig. 3c, d). **c** A rank correlation was performed to test if same genes have comparable BCV’s ex vivo and in vitro (Spearman’s rank correlation coefficient = 0.233, *p* value = 8.2 × 10^–45^). The point colors and sizes indicate the density of the plots and the sequencing depth of the genes (log 2 cpm), respectively. Source data are provided as a Source Data file

We next sought to investigate whether the five genetically diverse populations might produce a more diverse transcriptional profile under conditions that are different from that of the ex vivo conditions. We therefore cultured the pool samples from the five sub-compartments (containing ~5000 isolates, whose genomes have been sequenced in pools) under rich medium conditions and recorded the transcriptional profiles. Indeed, a multidimensional scaling plot revealed a greater diversity in the in vitro samples (Fig. 5a). To gain more detailed insights we also recorded the overall variance in the gene expression profiles (biological coefficient of variation (BCV)) across the different sub-populations ex vivo as compared to in vitro. The great majority of genes exhibited a comparable variance of expression (Fig. 5b). Nevertheless, there were significantly more in vitro expressed genes with a BCV ≥0.3686 (upper 10% of all BCVs) than ex vivo expressed genes (odds ratio 1.995, *p* value 2.4 × 10^–17^; Fisher’s exact test) (Fig. 5b). Clearly, most of the individual genes were variantly expressed to the same extent ex vivo as compared to in vitro (diagonal in Fig. 5c).

### CF habitat-specific transcriptional profile

**Fig. 6:**
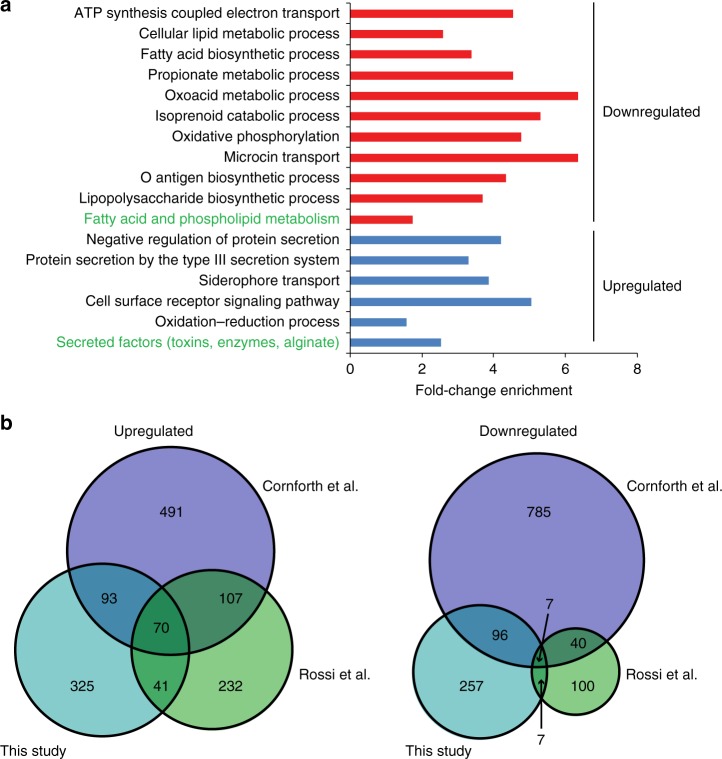
Cystic fibrosis (CF) habitat-specific transcriptional profile. Genes that were differentially expressed (log 2 fold change (FC) ≥1.5) in the ex vivo transcriptional profile compared to the in vitro condition were assigned to GO terms and PseudoCAP categories (green text). The enrichment factor is depicted in **a** and provides information on the proportion of regulated genes belonging to a functional category relative to the proportion of all genes (that were considered for differential expression analysis) belonging to that category. Categories with significantly enriched genes are depicted (hypergeometric test, adjusted *p* value ≤0.05), while fold-change enrichment is indicated at the *x* axis. Source data are provided as a Source Data file. **b** Differentially expressed genes (ex vivo vs. in vitro) identified in this study (log 2 FC ≥1.3, adjusted *p* value <0.05) were compared with those identified in two previous studies, which analyzed the *P. aeruginosa* transcriptional profile in CF sputum samples (Rossi et al.^23^ and Cornforth et al.^24^ (log 2FC ≥1.3, adjusted *p* value <0.05)). Lists of the overlapping genes are found in Supplementary Data 2

To identify and characterize the ex vivo transcriptional profile of *P. aeruginosa* during a chronic infection of the CF lung, we recorded differentially expressed genes between the ex vivo and the rich medium grown pooled samples across all five sub-compartments. Overall 699 genes were differentially expressed (log 2 fold change significantly higher than 1.5). The majority of them were up-regulated ex vivo when compared to laboratory conditions (433 genes, Supplementary Data 1). Figure 6a depicts the enriched expression of genes belonging to distinct functional groups during the ex vivo conditions. As described previously^23,24^, *P. aeruginosa* faces obviously antibiotic, oxidative, and osmotic stress in the microenvironment of a CF lung. Accordingly, genes that are linked to antibiotic resistance were up-regulated, for example, genes encoding the intrinsic β-lactamase AmpC^23^, the protease PfpI^36^, or *mexF*, encoding for components of the multi-drug efflux pump MexE-MexF-OprN^37^ (a list of all differentially regulated genes can be found as Supplementary Data 1). Furthermore, genes of the oxidative stress response such as *katA*, *katB*, *katN* (encoding for catalases), s*odB*, *sodM* (encoding a superoxide dismutase) or *ahpC*, *ahpF* (encoding hydroperoxide reductase subunits) were up-regulated^23^. In addition, the osmotic stress response genes *betA*, *betB*, and *betI* (involved in production of glycine-betaine) were expressed at increased levels ex vivo^23^. *Pseudomonas aeruginosa* adaptation to the microaerophilic conditions of the chronically infected CF lung is reflected in the up-regulation of denitrification genes (*narI*, *narH*, *narG*, *nosF*, *nosR*, and *norB*) as well as the lactate dehydrogenase genes (*lldA*, *lldD*, *lldP*). Furthermore, genes encoding NADH dehydrogenases *PA2691*, *nuoD*, *nuoG*, *nuoK*, *nuoL*, *nuoH* were down-regulated.

The ex vivo transcriptional profile also seems to reflect a low-energy, reduced-growth, and non-motile physiological state. Genes of the functional group of fatty acid metabolism (*fabD, fabI, fabG*, *accB*, *accC*, *accD*) as well as genes involved in motility and attachment (*pilA*, *pilG*, *pilS*) were down-regulated. Also, genes involved in amino acid biosynthesis were down-regulated in the microenvironment of the CF lung.

In the CF lung *P. aeruginosa* furthermore encounters an environment that is devoid of free iron. Thus, genes involved in the heme acquisition or transport (*hemA*, *K*, *hasR*, *hasD*, *phuR*, *phuT*) were expressed at higher levels ex vivo^20^. Additionally, we found two operons that are involved in iron transport and uptake, and that exhibited a higher expression ex vivo (*fox* operon and *fem* operon) and an up-regulation of the pyoverdine siderophore biosynthetic genes.

While many of the *pil* genes were down-regulated ex vivo, we found *algA* and *algD* were among the genes exhibiting the highest ex vivo expression^38^. In addition, genes encoding components of the type III secretion system (T3SS) were significantly up-regulated (*pscC*, *pscJ*, *pscI*, *pcrD*, *pcrH*, *pcrV*, *popB*, *popD*, *popN*). In accordance, the small regulatory RNA *rsmY* was down-regulated, thus promoting up-regulation of the T3SS. *RsmY* also positively controls the type VI secretion system, which was found to be expressed at lower levels ex vivo^39^.

Figure 6b demonstrates that especially among the up-regulated genes a high proportion (38.56%) have already been described previously to be up-regulated in the sputum of chronically infected patients^23,24^, whereas among the genes that were down-regulated in the CF lung environment the overlap was smaller (29.97%). A list of the overlapping genes among all three studies can be found in the Supplementary Data 2.

## Discussion

The success of the environmental bacterium *P. aeruginosa* as an opportunistic pathogen is mainly based on its very efficient adaptation to the changing and challenging conditions within the human host. The environmental pressure of the habitat of the CF lung produces adapted genetic *P. aeruginosa* variants that can be repeatedly isolated in chronically infected CF patients^20^. In this study, we found mutations in various combinations, especially in resistance conferring genes, highlighting the importance of parallel evolution of adaptive mutations. Several studies have shown that there are a number of bacterial traits that show the signature of within-patient directional selection in the CF environment^22,40^. In addition to genes that confer antibiotic resistance, multiple adaptive mutations arise, for example, in genes that encode for outer membrane components and iron scavenging systems^10,11,13,20,22,41^. The acquisition of different positively selected mutations drives genetic diversity. Accordingly, it has been demonstrated that *P. aeruginosa* CF isolates exhibit a very diverse array of phenotypes in vitro^9,42,43^. However, these mutations obviously do not sweep to fixation. Instead, in the CF lung diversifying lineages coexist for many years. A key factor for driving diversity despite directional selection seems to be the regional isolation of *P. aeruginosa* in different lung compartments^9^. In agreement, we also found a genetically diverse *P. aeruginosa* population structure in various compartments of an explanted CF lung. Obviously divergent evolution took place over many years as it has been estimated that between 1.1 and 5.5 SNPs per year in serially collected isolates can be detected^27,44,45^.

In this study, we also aimed to address the question of how, in addition to genetic diversity, the *P. aeruginosa* transcriptional response contributes to bacterial adaptation to the challenging environment of the CF lung during a chronic infection progress. We therefore recorded an ex vivo transcriptional profile from an explanted CF lung. Because the processing of the explanted CF lung took time, there is the possibility that the transcriptional response recorded in this study does not accurately reflect the in vivo transcriptional profile. Also, we obtained data form only one single explanted lung. Nevertheless, the genetic differences between the *P. aeruginosa* populations in the various sub-compartments of the explanted lung seem to not have had a large impact on the general ex vivo transcriptional response to the CF lung environment. This is interesting, as a common environment-driven transcriptional profile could contribute to the maintenance of genetic diversity in the CF population, because the genetic variants do not produce a phenotype that can be selected for or against. However, caution must be taken when considering that many mutations that arise in the CF environment might be neutral. The common transcriptional profile of the genetically diverse sub-populations in this study might also be explained by the fact that transcriptional profiling of populations obscures diversity among the individual isolates within the population. We observed a strong positive correlation between the frequency of SNPs in genome and cDNA sequencing reads, indicating that also low abundant clones within the population contribute to the overall transcriptional profile. Nevertheless, only transcriptional profiling on the single-cell level would unambiguously demonstrate that the transcriptional response in the CF environment is indeed independent of the genetic background.

The genetic variations that were found between *P. aeruginosa* isolates inhabiting the various sub-compartments might be expected to have an impact on the bacterial phenotype when grown under conditions different from the CF environment^46^. Indeed, we found that there were genes that were expressed at a higher level of diversity across the *P. aeruginosa* isolates from the five CF lung sub-compartments under rich medium conditions as compared to the ex vivo conditions. The acquisition of mutations in genes that are not neutral under rich medium conditions could account for this. However, alternatively, expression of those genes might be more critical in the ex vivo environment, whereas under rich medium conditions transcriptional control could be more relaxed.

An environment-induced trait that was originally triggered by an environmental cue might become fixed and thus be expressed constitutively in a population. It was previously demonstrated that the mRNA levels for genes controlled by the quorum-sensing systems were considerably lower in *P. aeruginosa* ex vivo transcriptomes than in in vitro transcriptomes^24^. Furthermore, genes encoding for efflux pumps associated with tolerance to aminoglycosides (*mexXY*) were found to be induced in multiple human infections^47^. Also *algU*, positively regulating the production of alginate, was found to be up-regulated ex vivo^24^. Strikingly, *mucA and mexZ* as well as *lasR* are among the most frequently mutated genes in *P. aeruginosa* isolated from chronic CF infections^22,26–28,38,48–50^. All three are encoding transcriptional regulators: LasR governs the production of quorum-sensing dependent virulence traits, while MucA encodes a negative regulator of alginate biosynthesis and MexZ a negative regulator of the MexXY efflux pump. In the *P. aeruginosa* population analyzed in this study, *lasR* and *mexZ* were mutated in all isolates, so that no differentially expressed MexZ- and LasR-governed genes were detected ex vivo. Nevertheless, in accordance with a previous study^24^ we found that the ex vivo transcriptional response of *P. aeruginosa* to the conditions encountered within the analyzed CF lung was dominated by an enhanced expression of the exopolysaccharide alginate. Thus, it seems that regulators of many of the ex vivo observed phenotypic traits are hot-spots of mutations during chronic CF lung infections and show the clear signature of within-patient selection. Albeit caution is required as we cannot estimate the extent to which the transcriptional response recorded here actually reflects the ex vivo transcriptional profile, the finding that commonly acquired genetic *P. aeruginosa* variants are found within global regulators, which govern CF environment relevant transcriptional responses, is interesting. This supports the hypothesis that phenotypic traits that are activated in the CF environment might eventually become fixed by the acquisition of mutations in respective global regulators.

## Methods

### Sample collection and histology

Tissue samples were taken from a freshly explanted lung of a CF patient undergoing double lung transplantation. The organ was transferred to the department of pathology for processing immediately after explantation at room temperature. Preparation times were kept as short as possible and samples were collected and transferred into RNAprotect within 30 min. Tissue containing dilated airways and mucopurulent secretions were sampled from five different locations of the left lung: large airways of the lung hilus and central and peripheral upper and lower lobe. To generate suitable samples for ex vivo RNASeq, one part of the tissue pieces was immediately homogenized and incubated for 10–15 min in RNAprotect reagent (Qiagen) at room temperature, centrifuged for 10 min at 10,000 rpm and the pellet was frozen at −80 °C. Another part of the tissue was homogenized in phosphate-buffered saline (PBS) to recover single *P. aeruginosa* isolates by streaking the homogenate on selective cetrimid-agar plates. Single colonies were picked and pooled samples of ~ 5000 single isolates were scraped off the plate. For the genomic approach, we sequenced ten single colonies following DNA extraction from subcultures in LB and two pools from five sub-compartments where DNA was extracted directly from the agar grown bacteria. In the transcriptomic dataset we included the five ex vivo samples and another five in vitro samples obtained from those cultured pools, which were also used for the genomic approach.

For histology, tissue specimens were fixed in 4% buffered formalin for 12–24 h. After fixation the tissue was embedded in paraffin according to standard histopathological protocols. The formalin-fixed and paraffin-embedded tissue was cut into sections of 4 µm thickness and stained with hematoxylin–eosin. Fluorescence in situ hybridization and analyses was conducted as follows: sections of 3 μm thickness were fixed on Super Frost slides (Thermo Scientific, USA), deparaffinized and air dried^51^. For dissolving components, FISH probes were preheated to hybridization temperature and 10 μl of the indocarbocyanine (Cy3)-labeled *P. aeruginosa*-specific probe (Biovisible, The Netherlands) mixture was applied on each section and covered airtight with a coverslip. After hybridization sections were uncovered and washed for 10 min. Sections were then rinsed with distilled water, air dried, and mounted with 4′, 6-diamidino-2-phenylindole (DAPI) Dura Tec (Zytomed Systems, Germany) and a coverslip. The specimens were imaged on digital microscope (BZ-9000, Keyence, Japan) using appropriate filters for the detection of DAPI and Cy3.

### Patient information

Tissue samples were obtained from a middle aged patient with CF (dF508/dF508) undergoing double lung transplantation at Hannover Medical School (MHH). The patient was chronically colonized with *P. aeruginosa* for 16 years. Experiments were performed in accordance with the regulations of the ethics committee at MHH (no. 2015-2700).

### RNA extraction, RNASeq, and transcriptomic analysis

Frozen cell pellets were used for ex vivo RNA extraction. The RNeasy Mini Kit (Qiagen) in combination with Qiashredder columns (Qiagen) was used according to the manufacturer’s instructions with some modifications. DNA was removed using the DNA-free^TM^ Kit (Thermo Fisher). To enrich the bacterial RNA MicrobeEnrich^TM^ Kit (Thermo Fisher) was used. For the in vitro RNA extraction the pools and single colonies recovered from the lung tissue were grown in LB media (inoculation with OD_600_ = 0.05) under shaking conditions (180 rpm) to early stationary phase (OD_600_ = 2) and RNA was extracted as described above. Obtained RNA was quality checked using the RNA Nano Kit (Agilent Technologies) on an Agilent Bioanalyzer 2100 (Agilent Technologies). The removal of ribosomal RNA was performed using the Ribo-Zero Bacteria Kit (Illumina) and cDNA libraries were generated with the ScriptSeq v2 Kit (Illumina). The samples were sequenced in single-end mode on an Illumina HiSeq 2500 device involving 50 cycles. Sequenced Illumina libraries produced ~37 million raw reads per sample (mean). Mapping was performed using a stampy pipeline^52^ using the PAO1 genome as a reference. Among the sequencing reads approximately 850,000 mapped to the PAO1 reference genome, indicating that >97% of all reads represented human transcripts. Differential gene expression analysis was performed with the R package edgeR (v.3.20.1)^53^ after removal of genes with <5 counts per million in any sample. Comparison between the ex vivo and in vitro condition was done using differential gene expression relative to a 1.5 fold-change threshold (TREAT; edgeR function glmTreat). For differential gene expression between the ex vivo sub-populations from the bronchus, central (ULC, LLC), and peripheral (ULP, LLP) region, as well as the bronchus, upper (ULC, ULP), and lower (LLC, LLP) region, an ANOVA-like test (edgeR function glmQLFTest) was used to increase the sensitivity to account for any compartment-dependent effects on the transcription level. Therefore, we used the samples from the different regions and treated them as replica. For functional enrichment, differential gene expression between central vs. peripheral, as well as upper vs. lower region was calculated (edgeR function glmQLFTest).

### Variance in the ex vivo and in vitro transcriptional profiles

To reduce the methodological bias, we first removed rRNAs, transfer RNAs, and the *ssrA* gene from the reads per gene data. Second, we accounted for a lower number of reads in the ex vivo (on average 850,000) compared to the in vitro samples (on average 6.7 billion), as a low number of reads might influence and increase the variance. Therefore, we subsampled the read numbers to a similar level using the R function rrarefy (R package vegan (v.2.5.2)). One sample with <500,000 reads (ex vivo LLP) was removed and the remaining samples were subsampled to the next sample with the lowest number of reads. To increase comparability between the samples, genes with less than 5 counts per million (cpm) in any sample were removed from the dataset and reads of the remaining 3525 genes were normalized using trimmed mean of *M* values. The BCV is the square root of the tag-wise dispersion (variation per gene), which has been calculated using the package edgeR for ex vivo and in vitro samples separately. Data were visualized as density plot using the R package ggplot2 (v.2.2.1)^54^. MDS plots were generated using the R function plotMDS (default settings) from the package edgeR.

### DNA extraction and sequencing analyses

DNA was extracted from cell pellets using the DNeasy Blood & Tissue Kit (Qiagen) and fragmented with the S2/E210 Focused-ultrasonicator (Covaris) to achieve 400-nt-long fragments. Library preparation was performed using NEBNext Ultra DNA Library Prep Kit for Illumina (NEB) according to the manufacturer’s instructions. The samples were sequenced on an Illumina HiSeq 2500 device (2 × 150 bp pair-ended reads). Raw reads were mapped to the PAO1 reference genome as described for RNASeq mapping. After quality filtering of specific nucleotide variations, each SNP position in the 50 isolates were checked in the integrative genome viewer (IGV) (v.2.3.98). To correlate the SNPs of the single isolate with those of the deep pool sequencing, every SNP position as identified in the genomes of the single isolates was checked in IGV. For all SNP positions the ratio of mutated isolates to all isolates was calculated. This ratio was then correlated to the frequencies of mutated reads in the corresponding pools. The mean value from two pools was used for analyses. Furthermore, the mutation frequencies in the cDNA samples were correlated to the mutation frequencies of the pools and single isolates (per compartment). Therefore, the SNP positions found in the pool genomes were checked in IGV in the cDNA samples in order to calculate the SNP frequencies (cut-off: 20 cDNA reads). Correlation coefficients and corresponding *p* values were performed using Pearson’s correlation (R function cor.test). All *p* values were corrected using the false discovery rate.

### Phylogenetic tree

The genomic DNA read files (fastq) were de novo assembled using SPAdes (v.3.10.1)^55^ with default settings and the “careful” option. From the output we used the “contigs.fasta” files for annotation with Prokka (v.1.12)^56^ with the options “metagenome” and “compliant”. From the gff output files a pan genome was created by Roary (v.1.007002)^57^, which creates a multi-FASTA alignment of all of the core genes using PRANK^58^. The tool FastTree (v.2.1) was used to calculate a phylogenetic tree. The phylogenetic tree was visualized with the iTOL online tool.

### Reporting summary

Further information on research design is available in the Nature Research Reporting Summary linked to this article.

## Supplementary information

Supplementary Information

Description of Additional Supplementary Files

Supplementary Data 1

Supplementary Data 2

Reporting Summary

Source Data

## Data Availability

The raw sequencing results of the whole-genome sequencing analysis have been deposited into the NCBI Sequence Read Archive under SRA accession number SRP158462. The raw RNASeq data have been deposited into NCBI Gene Expression Omnibus under GenBank accession number GSE119356.
