# Supplementary material for: Genetically diverse Pseudomonas aeruginosa populations display similar transcriptomic profiles in a cystic fibrosis explanted lung
Source: Nat Commun. 2019 Jul 30;10:3397. doi: 10.1038/s41467-019-11414-3 (PMC6667473; doi:10.1038/s41467-019-11414-3)
Supplement: Supplementary file 1 — Supplementary Information [file 41467_2019_11414_MOESM1_ESM.pdf]

Supplementary information

Genetically diverse Pseudomonas aeruginosa populations display similar transcriptomic profiles in a cystic fibrosis explanted lung.

Kordes *et al*.

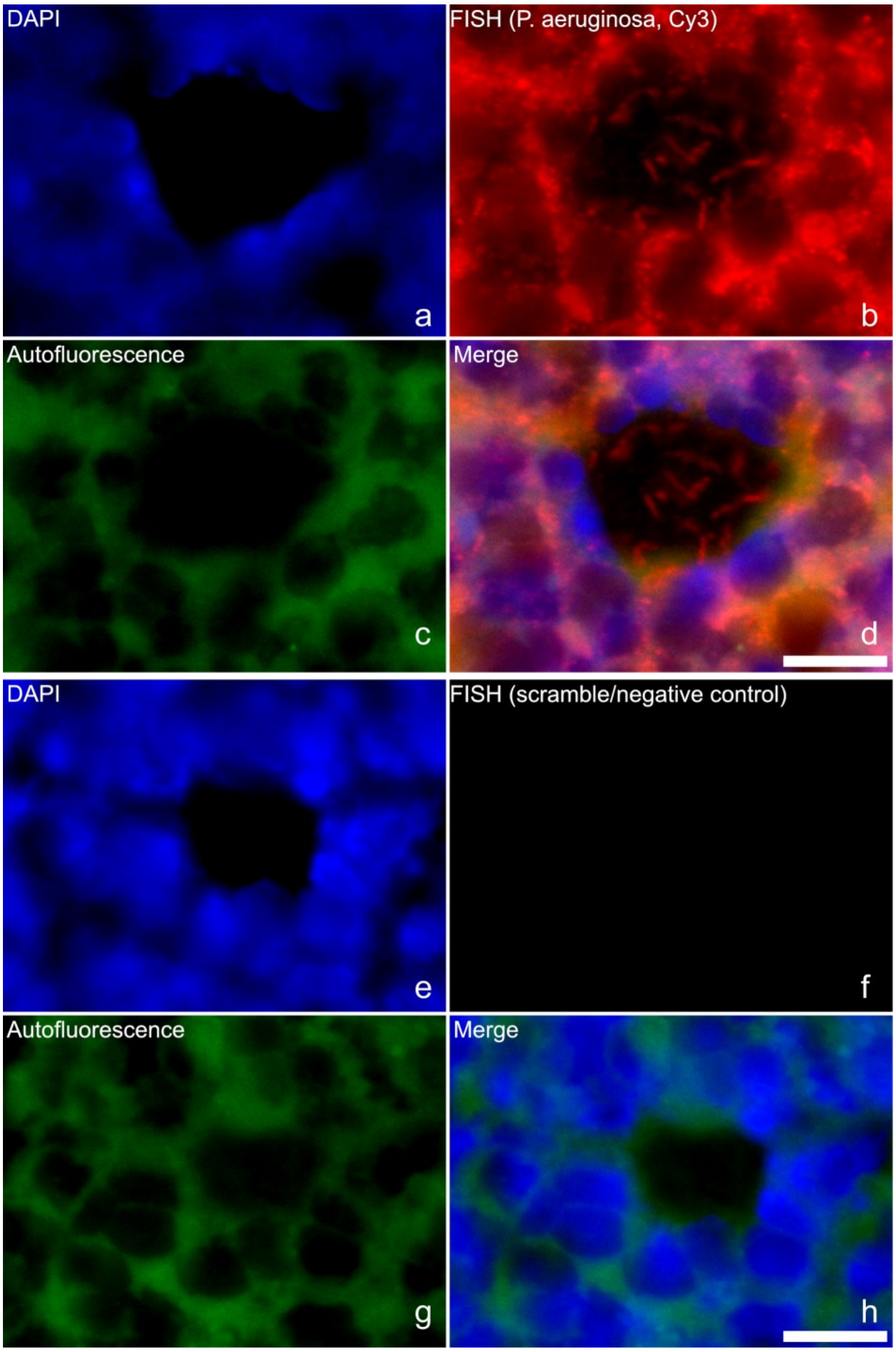

Supplementary Figure 1 Histological analysis of the CF-lung. (a-d) Bacterial colonies can be visualized by fluorescence *in situ* hybridization with Cy3 labelled oligonucleotide probes targeted against sequences on the 16S ribosomal RNA specific for *P. aeruginosa*. Nuclear counterstain by DAPI. Green shows autofluorescence in the FITC filter. (e-h) Bacterial colonies in the negative control *in situ* hybridization with Cy3 labelled scrambled oligonucleotide targeting no specific sequence. Nuclear counterstain by DAPI. Green shows autofluorescence in the FITC filter. Scalebars are 10 µm.

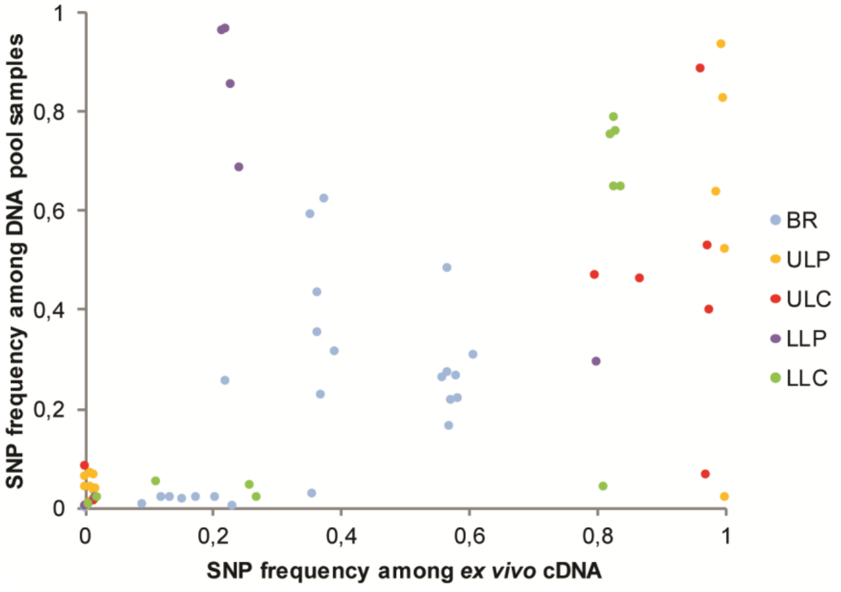

Supplementary Figure 2 Correlation of the mutation frequencies of in vivo cDNA samples (RNASeq) and DNA sequences of genome pool samples. SNP frequencies for each SNP position from cDNA sequencing (RNAseq) and the corresponding frequencies of DNA sequencing of the genome pool samples per compartment are plotted (cDNA coverage was at least 50 reads and at least 20 reads for the LLP sample). Source data are provided as a Source Data file

The correlation between cDNA and genome SNPs increased with higher cDNA coverage. In the BR, ULP and ULC sample we found a correlation of 0.6497, 0.9599 and 0.8929, respectively (with at least 100 reads, adjusted p value < 0.05). In the LLP sample we found a correlation of 0.1272 in SNP positions with a minimum of 20 cDNA reads (adjusted p value = 0.78). In the LLC sample we found a correlation of 0.8335 in SNP positions with a minimum of 20 cDNA reads (adjusted p value < 0.05).

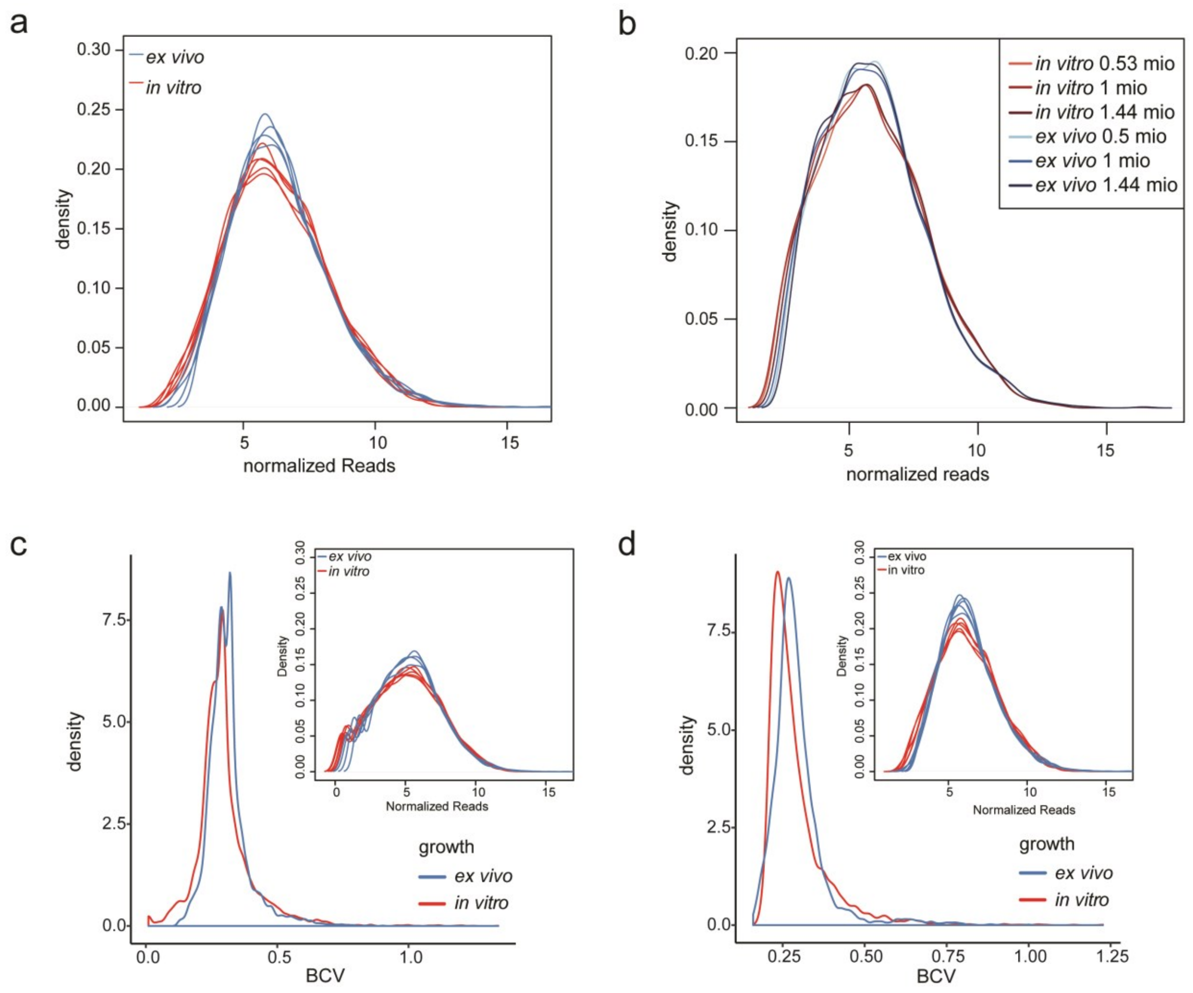

Supplementary Figure 3 Effect of data processing on sample distribution. Distribution of normalized reads (a and inset plots of c and d) and the biological coefficient of variation (c and d). (b) Effect of subsampling on sample distribution. An *in vivo* and *in vitro* sample (ULP) was subsampled to 0.53 mio (*in vivo* ULP reads), 1 mio, and 1.44 mio reads, respectively. y axis: density; x axis: normalized log2 reads (cpm and TMM using edgeR). Remaining genes after filtering: 4162 (0.53 mio reads); 4273 (1 mio reads); 4166 (1.44 mio reads). (c) Data were subsampled as shown in Fig. 5a, but without additional read filter. (d) Data were not subsampled, but reads were filtered by removing genes with less than 5 normalized reads (cpm) in all samples. Source data are provided as a Source Data file.

Supplementary Table 1 Differentially expressed genes in ANOVA-like analysis.

	log2FC			adjusted p value
	BR versus peripher	BR versus central	peripher versus central	
PA3305.1,phrS	3.8	2.0	-1.8	0.019
PA4587,ccpR	3.6	1.7	-1.9	0.019
PA3871	3.2	1.6	-1.6	0.030
PA3877,narK1	3.3	1.6	-1.7	0.030
PA2119	2.9	1.8	-1.1	0.030
PA2753	4.2	1.6	-2.6	0.030
PA3613	2.7	1.6	-1.1	0.030
PA3874,narH	3.3	1.4	-1.9	0.033
PA3872,narI	3.0	1.3	-1.7	0.033
PA3875,narG	3.4	1.5	-1.9	0.033
PA5027	3.2	1.9	-1.3	0.033
PA4328	3.1	1.9	-1.2	0.033
PA1546,hemN	3.0	1.5	-1.6	0.033
PA3337,rfaD	3.3	1.5	-1.8	0.034
PA3870,moaA1	3.0	1.2	-1.8	0.036

Supplementary Table 2 Functional enrichment (GO biological process) of 100 genes with lowest p values and log2FC of ≥ 1

GO term	conditions	regulation	FC enrichment	adjusted p value
siderophore transport	peripher vs central	up	13.5	0.00124
pyoverdine biosynthetic process	peripher vs central	up	18.5	0.00994
protein secretion by the type III secretion system	peripher vs central	up	10.2	0.00994
cell surface receptor signaling pathway	peripher vs central	up	13.0	0.01885
arginine deiminase pathway	peripher vs central	down	36.4	0.00003
response to stress	peripher vs central	down	13.0	0.00072
nitrogen compound metabolic process	peripher vs central	down	6.7	0.01296
nitrate metabolic process	peripher vs central	down	36.4	0.01296
pyoverdine biosynthetic process	upper vs lower	up	26.0	0.00002
siderophore transport	upper vs lower	up	13.7	0.00008
cell surface receptor signaling pathway	upper vs lower	up	18.2	0.00008
heme transport	upper vs lower	up	15.6	0.01263
negative regulation of protein secretion	upper vs lower	up	13.7	0.01585
response to stimulus	upper vs lower	up	4.2	0.03492
regulation of iron ion transport	upper vs lower	up	18.2	0.04810
