# Supplementary material for: Genetically diverse Pseudomonas aeruginosa populations display similar transcriptomic profiles in a cystic fibrosis explanted lung
Source: Nat Commun. 2019 Jul 30;10:3397. doi: 10.1038/s41467-019-11414-3 (PMC6667473; doi:10.1038/s41467-019-11414-3)
Supplement: Supplementary file 2 — Description of Additional Supplementary Files [file 41467_2019_11414_MOESM2_ESM.pdf]

Description of Additional Supplementary Files

File Name: Supplementary Data 1

Description: Differential gene expression ex vivo pool vs. in vitro pool. Ex vivo derived transcriptional profiles from five sub-compartments were treated as one replica as well as the five corresponding pool samples (containing approx. 5000 isolates) cultured under rich medium conditions in vitro. Genes that were differentially expressed (log2 fold change ≥ 1.5) in the ex vivo transcriptional profile compared to the in vitro condition are listed.

File Name: Supplementary Data 2

Description: Differentially expressed genes (ex vivo vs. in vitro) identified in this and two other studies. Each Dataset represent the comparison between an ex vivo and in vitro condition. The overlay of differentially expressed genes (up- and downregulated) of this study (log2FC ≥1.3, adjusted p value <0.05) and two previous studies, which analyzed the P. aeruginosa transcriptional profile in CF sputum samples are listed. Dataset X = Cornforth et al. 1 (log2FC ≥1.3, adjusted p value <0.05, CF sputum vs in vitro), Dataset Y = this study, Dataset Z = Rossi et al. 2 (Cluster I (Lungs) vs Cluster II (Lab. exp.) and Cluster I (Lungs) vs Cluster III (Lab. stat.), log2FC ≥1.3, adjusted p value <0.05)).
