# Supplementary material for: Genetically diverse Pseudomonas aeruginosa populations display similar transcriptomic profiles in a cystic fibrosis explanted lung
Source: Nat Commun. 2019 Jul 30;10:3397. doi: 10.1038/s41467-019-11414-3 (PMC6667473; doi:10.1038/s41467-019-11414-3)
Supplement: Supplementary file 5 — Reporting Summary [file 41467_2019_11414_MOESM5_ESM.pdf]

nature research

Corresponding author(s): Susanne Häussler

Last updated by author(s): Jun 21, 2019

Reporting Summary

Nature Research wishes to improve the reproducibility of the work that we publish. This form provides structure for consistency and transparency in reporting. For further information on Nature Research policies, see Authors & Referees and the Editorial Policy Checklist.

Statistics

For all statistical analyses, confirm that the following items are present in the figure legend, table legend, main text, or Methods section.

n/a	Confirmed	
☐	☒	The exact sample size (*n*) for each experimental group/condition, given as a discrete number and unit of measurement
☐	☒	A statement on whether measurements were taken from distinct samples or whether the same sample was measured repeatedly
☒	☐	The statistical test(s) used AND whether they are one- or two-sided<br>*Only common tests should be described solely by name; describe more complex techniques in the Methods section.*
☒	☐	A description of all covariates tested
☐	☒	A description of any assumptions or corrections, such as tests of normality and adjustment for multiple comparisons
☐	☒	A full description of the statistical parameters including central tendency (e.g. means) or other basic estimates (e.g. regression coefficient) AND variation (e.g. standard deviation) or associated estimates of uncertainty (e.g. confidence intervals)
☐	☒	For null hypothesis testing, the test statistic (e.g. *F*, *t*, *r*) with confidence intervals, effect sizes, degrees of freedom and *P* value noted<br>*Give P values as exact values whenever suitable.*
☒	☐	For Bayesian analysis, information on the choice of priors and Markov chain Monte Carlo settings
☒	☐	For hierarchical and complex designs, identification of the appropriate level for tests and full reporting of outcomes
☒	☐	Estimates of effect sizes (e.g. Cohen's *d*, Pearson's *r*), indicating how they were calculated

Our web collection on statistics for biologists contains articles on many of the points above.

Software and code

Policy information about availability of computer code

Data collection	No software was used to collect data.
Data analysis	Mapping was performed using a stampy pipeline using the PAO1 genome as a reference.<br>Differential gene expression analysis between the in vivo and in vitro condition was performed with the R package DESeq2 (v.1.18.1).<br>Differential gene expression analysis was performed with the R package edgeR (v.3.20.1)53 after removal of genes with less than 5 counts per million in any sample. Comparison between the in vivo and in vitro condition was done using differential gene expression relative to a 1.5 fold change threshold (TREAT; edgeR function glmTreat). For differential gene expression between the in vivo sub-pupulations from the bronchus, central (ULC, LLC) and peripheral (ULP, LLP) region as well as the bronchus, upper (ULC, ULP) and lower (LLC, LLP) region, an ANOVA-like test (edgeR function glmQLFTest) was used to increase the sensitivity to account for any compartment-dependent effects on transcription level. Therefore, we used the samples from the different regions and treated them as replica. For functional enrichment, differential gene expression between central versus peripher, as well as upper versus lower region was calculated (edgeR function glmQLFTest).<br>To reduce the methodological bias, we first removed rRNAs, tRNAs, and the ssrA gene from the reads per gene data. Second, we took account for a lower number of reads in the in vivo (on average 850 000) compared to the in vitro samples (on average 6.7 billion), as a low number of reads might influence and increase the variance. Therefore we subsampled the read numbers to a similar level using the R function rrarefy (R package vegan (v.2.5.2)). One sample with less than 500 000 reads (in vivo LLP) was removed and the remaining samples were subsampled to the next sample with the lowest number of reads. To increase comparability between the samples, genes with less than 5 counts per million (cpm) in any sample were removed from the dataset and reads of the remaining 3525 genes were normalized using trimmed mean of M-values (TMM). The biological coefficient of variation is the square root of the tag wise dispersion (variation per gene), which has been calculated using the package edgeR for in vivo and in vitro samples separately. Data were visualized as density plot using the R package ggplot2 (v.2.2.1)54. MDS plots were generated using the R function plotMDS (default settings) from the package edgeR.<br><br>For SNP detection samtools (v.0.1.19) and parSNP (v.1.2) were used with the PAO1 genome as a reference and default parameters.<br>After quality filtering of specific nucleotide variations each SNP position in the 50 isolates were checked in the integrative

genome viewer (IGV) (v.2.3.98). Correlation coefficients and corresponding p values were performed using Pearson correlation (R function cor.test). All p values were corrected using the false discovery rate (FDR).
The genomic DNA read files (fastq) were de novo assembled using SPAdes (v.3.10.1) with default settings and the 'careful' option. From the output we used the 'contigs.fasta' files for annotation with Prokka (v.1.12) with the options 'metagenome' and 'compliant'. From the gff output files a pan genome was created by Roary (v.1.007002) which creates a multi-FASTA alignment of all of the core genes using PRANK. The tool FastTree (v.2.1) that is implemented in Roary, was used to draw a phylogenetic tree. The phylogenetic tree was visualized with the iTOL online tool.

For manuscripts utilizing custom algorithms or software that are central to the research but not yet described in published literature, software must be made available to editors/reviewers. We strongly encourage code deposition in a community repository (e.g. GitHub). See the Nature Research guidelines for submitting code & software for further information.

Data

Policy information about availability of data

All manuscripts must include a data availability statement. This statement should provide the following information, where applicable:

- Accession codes, unique identifiers, or web links for publicly available datasets
- A list of figures that have associated raw data
- A description of any restrictions on data availability

The raw sequencing results of the whole genome sequencing analysis have been deposited into the NCBI Sequence Read Archive under SRA accession number SRP158462 [https://www.ncbi.nlm.nih.gov/sra/SRP158462]. The raw RNA-Seq data have been deposited into NCBI Gene Expression Omnibus under GenBank accession number GSE119356 [https://www.ncbi.nlm.nih.gov/geo/query/acc.cgi?acc=GSE119356].

Field-specific reporting

Please select the one below that is the best fit for your research. If you are not sure, read the appropriate sections before making your selection.

☒ Life sciences ☐ Behavioural & social sciences ☐ Ecological, evolutionary & environmental sciences

For a reference copy of the document with all sections, see nature.com/documents/nr-reporting-summary-flat.pdf

Life sciences study design

All studies must disclose on these points even when the disclosure is negative.

Sample size	Samples were taken from a transplanted lung of one patient at five sites: main bronchus (BR), upper lobe central (ULC) and peripheral (ULP), and lower lobe central (LLC) and peripheral (LLP). From these five sites RNA was extracted ex vivo (from lung tissue) and in vitro (LB-culture), respectively. Recovery of single isolates and pools of P. aeruginosa was performed by plating on cetrimid-agar. Per region of the lung 10 single isolates and two pools of approx. 5 000 colonies were collected for genome sequencing.
Data exclusions	For measurement of variances of gene expression in vitro and in vivo (Fig. 5) the in vivo LLP transcriptional profile has been removed prior analysis because less than 500 000 reads were obtained.
Replication	We correlated genome sequencing of the single isolates and pools and saw overall good correlation (Fig. 3). Reproducibility of ex vivo RNA-Seq is not possible because all samples from the fresh transplanted lung were used directly.
Randomization	The goal was to analyze the in vivo transcriptional profile of different locations in the CF-lung. Thus five sites were chosen and samples were collected as described in the sample size section. 10 Single isolates per region were picked randomly from plate for genome sequencing.
Blinding	Since there were no group allocation of any of our samples blinding was not relevant for this study. Data analysis was automated and no bias toward any region or sample type was possible.

Reporting for specific materials, systems and methods

We require information from authors about some types of materials, experimental systems and methods used in many studies. Here, indicate whether each material, system or method listed is relevant to your study. If you are not sure if a list item applies to your research, read the appropriate section before selecting a response.

Materials & experimental systems

n/a	Involved in the study	
☒	☐	Antibodies
☒	☐	Eukaryotic cell lines
☒	☐	Palaeontology
☒	☐	Animals and other organisms
☒	☐	Human research participants
☒	☐	Clinical data

Methods

n/a	Involved in the study	
☒	☐	ChIP-seq
☒	☐	Flow cytometry
☒	☐	MRI-based neuroimaging
